# Emergency laparoscopic resection of spontaneous rupture of hepatocellular carcinoma: A case report

**DOI:** 10.1016/j.ijscr.2019.11.055

**Published:** 2019-12-03

**Authors:** Allim Khairuddin, Guang Hong Ong, Jun Sam Tan, Syamim Johan, Vee Chuan Hoe, Mohd Sharifudin Sharif, Firdaus Hayati

**Affiliations:** a Department of Surgery, Queen Elizabeth Hospital, Ministry of Health Malaysia, Kota Kinabalu, Sabah, Malaysia; b Gleneagles Kota Kinabalu, Kota Kinabalu, Sabah, Malaysia; c Department of Surgery, Faculty of Medicine and Health Sciences, Universiti Malaysia Sabah, Kota Kinabalu, Sabah, Malaysia

**Keywords:** Laparoscopy, Hepatectomy, Hepatocellular carcinoma, Rupture, Case report

## Abstract

**Introduction:**

Laparoscopic liver resection is currently performed as a therapeutic modality in hepatocellular carcinoma (HCC). In an emergency setting such as bleeding or rupture, however it has not been well documented.

**Presentation of case:**

We describe a 55-year-old lady who presented to the emergency department with epigastric pain and symptoms of anaemia for one day duration. She was normotensive but tachycardic. Blood investigations revealed haemoglobin level of 6.5 g/dL and serum alpha-fetoprotein of 3136 g/dL. Contrast enhanced computed tomography scan revealed ruptured HCC of segment 2 and 3. She underwent emergency laparoscopic resection of the ruptured HCC. The postoperative recovery was uneventful and she was discharged well on postoperative day 7. Histology confirmed a 10 cm ruptured HCC with 3 mm tumour-free resection margin.

**Discussion:**

Ruptured HCC is associated with a high mortality rate of 25–75 %. Traditional treatment involves initial stabilization and hemostasis through transarterial embolization followed by staged hepatic resection. However, laparoscopic liver resection has been shown to be superior than open surgery in terms of postoperative outcomes.

**Conclusion:**

Laparoscopic resection of bleeding HCC is achievable and can be considered in the treatment algorithm of selected patients.

## Highlights

• Ruptured hepatocellular carcinoma is associated with a significant morbidity and very high mortality rate.
• Epigastric pain with hypovolaemic shock in addition to a raised serum alpha-fetoprotein can be suggestive of ruptured hepatocellular carcinoma.
• One staged laparoscopic surgery can be considered among hemodynamically stable candidates with no ongoing bleeding and good functional liver reserve.

## 1 Introduction

Hepatocellular carcinoma (HCC) is the fifth most common cancer in the world [1]. One of the life-threatening complications of HCC is rupture of the tumour with intraperitoneal haemorrhage. In most instances, a staged liver resection is advocated as the preferred definitive treatment [2,3] However, 1-stage emergency liver resection can still be considered in stabilized patients with an easily accessible tumour and good functional liver reserve [3]. Laparoscopic liver resection was first reported by Gagner et al. in 1992 [2]. However, because of its complexity, laparoscopic liver resection was not widely adopted and the first large case series was only reported 8 years later in 2000 by Cherqui et al. in 30 patients [4]. Since then with the introduction of various new and improved laparoscopic devices, laparoscopic liver resection has been increasingly adopted worldwide and numerous series have been published in the literature [[5], [6], [7]].

Laparoscopic liver resection has been shown to be superior than open surgery in terms of postoperative outcomes such as shorter duration of hospital stay and decreased pain [[5], [6], [7]]. It has also been shown to be safe with blood transfusion rates, blood loss, postoperative morbidity and mortality rates that are similar to if not lower than those with open surgery [[5], [6], [7], [8]]. Numerous investigators have also confirmed its effectiveness for treating malignant lesions such as HCC, reporting similar tumor-free margins and survival rates compared with open resection [[8], [9], [10]]. Spontaneously ruptured HCC has been reported to occur in 3%–15% of HCC patients and is associated with a high mortality rate of 25–75 % due to liver failure [2]. In this study we report a case of spontaneously ruptured HCC treated by early laparoscopic resection. This work has been reported in line with the SCARE criteria [11].

## 2 Case report

**Fig. 1:**
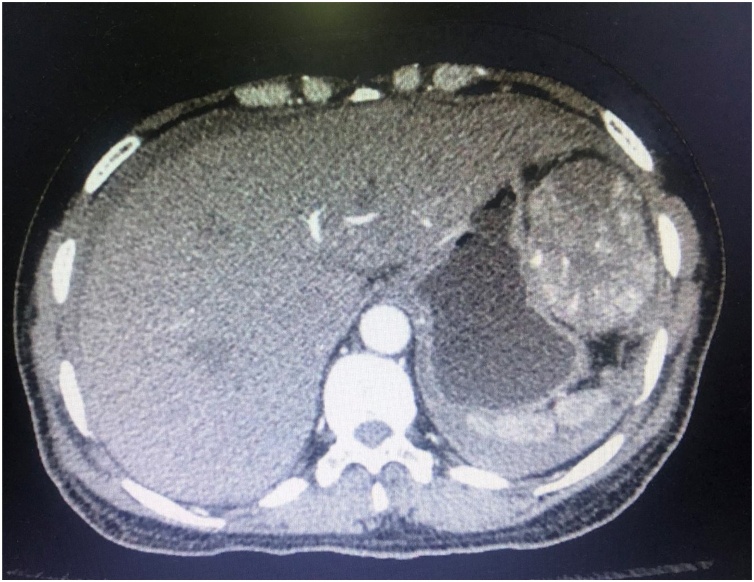
Contrast enhanced computed tomography demonstrated segment 2 and 3 liver lesion with wall defect and perihepatic hematoma.

A 55-year-old female with no known medical illness presented to the emergency department with epigastric pain and symptoms of anaemia for one day duration. On physical examination, she was normotensive with tachycardia and tenderness over the epigastric region. Her haemoglobin level was 6.5 g/dL. Her serum biochemistry panel showed evidence of acute kidney injury but liver function tests was unremarkable. She was transfused with 1U of blood and repeated haemoglobin level was 5.8 g/dL. The serum alpha-fetoprotein level was elevated at 3136 g/dL. She was transfused another 2 U of blood. Computed tomography scan performed on the same day showed a large liver mass in segment 2 and 3 of the left liver lobe with multiple areas of wall defect associated with layering of free fluid surrounding the liver suggestive of ruptured liver mass (Fig. 1). The patient was diagnosed with ruptured HCC.

**Fig. 2:**
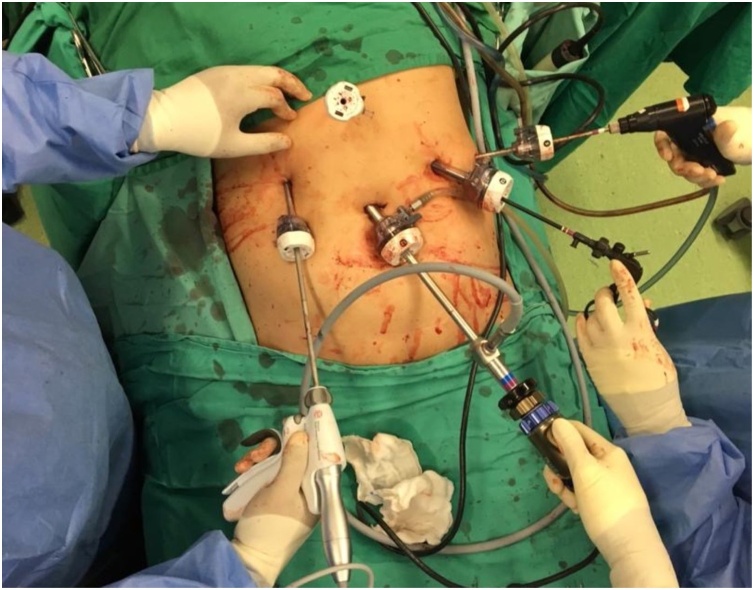
Port placements during the surgery.

**Fig. 3:**
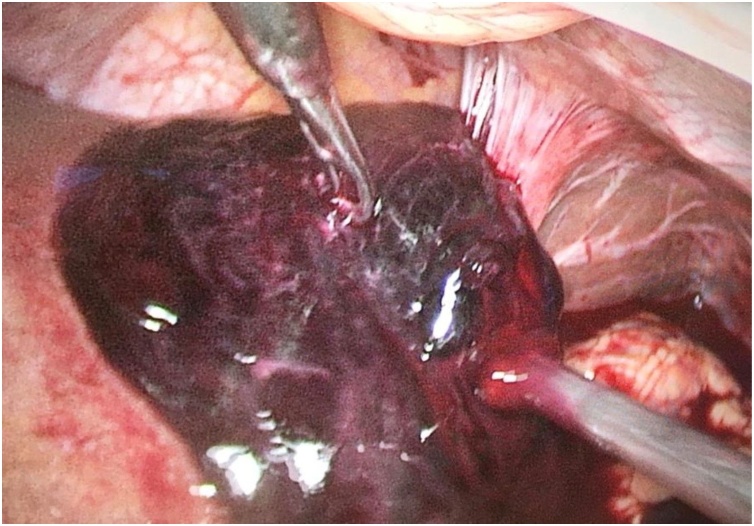
Laparoscopic view demonstrating hepatoma involving segment 2 of liver prior to resection.

She underwent emergency laparoscopic resection of the ruptured HCC approximately 30 h after her presentation to the hospital. The procedure was performed by use of a 12 mm supraumbilical port for the laparoscope, a 12 mm epigastric port, and 3−5 mm ports (Fig. 2). Laparoscopy confirmed a ruptured liver tumour with 2 L of hemoperitoneum (Fig. 3A and 3B). The tumour margins were confirmed by intraoperative ultrasonography and resection margins marked by cautery. Pringles manoeuvre was done using an umbilical tape and nasogastric tube for 15 min. Parenchymal transection was performed with Harmonic scalpel. A 5-cm incision over the left subcostal was made to extract the specimen through a specimen bag. After completion of the resection, a thorough washout of the entire abdominal cavity was done. The operation time was 180 min. The patient’s postoperative recovery was uneventful and she was discharged on postoperative day 7. Histology confirmed a 10 cm ruptured HCC with 3 mm tumour-free resection margin.

## 3 Discussion

Ruptured HCC is associated with a high mortality rate of 25–75 % [2]. The definite treatment of ruptured HCC has not been well documented. Initial management concentrates on adequate resuscitation with hemostasis. Presently, the most commonly adopted approach would be initial stabilization and hemostasis through transarterial embolization followed by staged hepatic resection [12]. In patients who are stable with no clinical or radiologic evidence of continuous bleeding, conservative management without hemostatic procedure may be advocated [13]. To date, open surgical hemostasis is reserved as a second line treatment in the event of failed transarterial embolization [2]. Liver resection is the only curative option for ruptured HCC. Proponents of 1-stage surgery argue that a delay in resection after hemostasis may compromise the resection rate because of tumor dissemination [2]. However, given the high inpatient mortality rates of 16.5%–100% associated with emergency resection, most authors today advocate staged liver resection, which is associated with inpatient mortality rates up to 9 % [2]. The poor outcome associated with emergency surgery is likely related to the unclear liver functional reserve and the high surgical risk in patients with hypovolemic shock [2]. Nonetheless, early 1-stage surgery may still be considered in highly selected patients who are hemodynamically stable with no ongoing bleeding and good functional liver reserve.

Laparoscopic liver resection is now well accepted in the treatment of HCC because it offers the usual benefits of minimally invasive surgery and has been shown to have oncologic outcomes similar to open resection [6,[8], [9], [10]]. However, its use in the treatment of ruptured HCC has not been well documented. In general, the use of laparoscopy in the setting of hemoperitoneum and shock has been limited because of concerns about visibility, exposure, and prolonged operating times [2]. In the study by Belgaumkar et al., 3 patients, 2 with ruptured HCC and 1 with ruptured hepatic adenoma, underwent successful laparoscopic liver resection [2]. The 3 patients underwent surgery 4 days, 12 days, and 21 days after presentation. This report shows that in a highly selected patient who can be hemodynamically stabilized and has no evidence of continuous active bleeding, good liver function, and easily accessible tumour, early laparoscopic liver resection can be considered a therapeutic option.

## 4 Conclusion

In conclusion, laparoscopic resection of ruptured HCC is feasible and should be considered in the treatment algorithm of selected patients who have been well stabilized based on preoperative liver function, location and the size of HCC.

## Sources of funding

The study did not receive any funding.

## Ethical approval

No ethical clearance required as it only involves case report.

## Consent

Written informed consent was obtained from the patient.

## Authors’ contributions

Involvement in managing the patient – AK, GHO, MSS.

Data collection – AK, JST, SJ.

Literature review – JST, VCH.

Manuscript editing – FH.

## Registration of research studies

No ethical clearance required as it only involves case report.

## Guarantor

Firdaus Hayati

## Provenance and peer review

Editorially reviewed, not externally peer-reviewed.

## Declaration of Competing Interest

The authors have no conflict of interests to declare.
